# Polymorphisms of Drug-Metabolizing Enzymes and Transporters Contribute to the Individual Variations of Erlotinib Steady State Trough Concentration, Treatment Outcomes, and Adverse Reactions in Epidermal Growth Factor Receptor–Mutated Non-Small Cell Lung Cancer Patients

**DOI:** 10.3389/fphar.2020.00664

**Published:** 2020-05-08

**Authors:** Dehua Liao, Zhigang Liu, Yongchang Zhang, Ni Liu, Dunwu Yao, Lizhi Cao, Yun Chen, Yilan Fu, Nong Yang, Daxiong Xiang

**Affiliations:** ^1^Institute of Clinical Pharmacy, Second Xiangya Hospital, Central South University, Changsha, China; ^2^Department of Pharmacy, Hunan Cancer Hospital, Changsha, China; ^3^The Cancer Center of The Fifth Affiliated Hospital of Sun Yat-sen University, Phase I Clinical Trial Laboratory, The Fifth Affiliated Hospital, Sun Yat-sen University, Zhuhai, China; ^4^Lung Cancer and Gastrointestinal Unit, Department of Medical Oncology, Hunan Cancer Hospital, Changsha, China

**Keywords:** erlotinib, non-small cell lung cancer, polymorphism, drug-metabolizing enzymes, transporters, steady state trough concentration

## Abstract

**Background:**

Erlotinib is presently the first line treatment for non-small cell lung cancer (NSCLC) with epidermal growth factor receptor (EGFR) active mutation. An increasing number of evidences show that the treatment efficacy and toxicities are considerably heterogeneous among individuals. Hence, it is necessary to find biological predictors for further individualized treatment of erlotinib in NSCLC patients.

**Methods:**

Our present study enrolled 87 cases of NSCLC patients who had been administrated erlotinib with a fixed dose (150 mg/d). Eleven polymorphisms in seven genes of drug-metabolizing enzymes and transporters were genotyped and the steady state trough concentrations were also determined.

**Results:**

There were significant variances in the steady-state erlotinib trough plasma concentrations, ranging from 315.6 ng/ml to 4479.83 ng/ml. Erlotinib steady state trough concentration was remarkably lower in current smoking patients. The steady state trough concentration of GG in rs1048943 of CYP1A1 was significantly higher than that of AA allele carriers. The polymorphism of CYP1A2 was significantly associated with the severity of skin rash, and the development of diarrhea was associated with SNPs in ABCB1 and CYP3A5. We also observed that GG allele in CYP1A1 was accompanied with a longer PFS in our study.

**Conclusion:**

A large variability of erlotinib steady state trough concentration was found among Chinese Han population. SNPs in CYP1A1 appeared to influence the steady state trough concentration of erlotinib. Correlation between CYP1A2 polymorphisms and severity of skin rash was observed, together with the correlation between the development of diarrhea and SNPs in ABCB1 and CYP3A5.

## Introduction

Non-small cell lung cancer (NSCLC) is a very common carcinoma, accounting for 80%–85% of lung cancer cases, and also the leading cause of cancer-related deaths worldwide. (Maemondo et al., 2010; Novello et al., 2016). The management of treatment for NSCLC could be optimized by using pharmacogenetic testing (Hamada et al., 2012). Numerous studies have showed that aberrant epidermal growth factor receptor (EGFR) pathway is a driving gene of NSCLC cancerogenesis, playing a considerable role in the process of malignancies, such as cell proliferation, differentiation and migration (Doerks et al., 2002; Bardelli et al., 2003). It was reported that the EGFR tyrosine kinase inhibitors (TKIs) were effective in patients with EGFR sensitive mutations (Mok et al., 2009; Mitsudomi et al., 2010).

Erlotinib is an orally administered, reversible TKI indicated as first-line treatment for advanced NSCLC in patients with activating EGFR mutations and as second- and third-line treatment in patients with EGFR wild-type NSCLC (Timmers et al., 2015). The most common activating mutations in EGFR are the exon 19 deletion and the exon 21-point mutation L858R (Tournier et al., 2017). Compared with standard platinum doublet therapy, erlotinib showed dramatically improved the long-term survival rate and clinical response of EGFR sensitive mutation NSCLC. Despite of its striking efficacy, however, real-world clinical evidence demonstrated considerable individual variability in both therapeutic efficacy and toxicity (Riely et al., 2009; Gainor and Shaw, 2013). Suboptimal response and response failure to the treatment are also observed in clinical treatment. In addition, most of the patients experienced drug-related adverse events (such as skin rash and diarrhea) with fixed dose of erlotinib during their treatment course (Endo-Tsukude et al., 2018).

Research in advanced NSCLC has since focused on finding biological predictors of response and adverse events allowing for treatment optimization. More evidences showed that inter-patient variability in drug response and adverse events which reflect the systemic levels or intracellular concentrations of the drug (Decosterd et al., 2015). Absorption, distribution, and metabolism are important pharmacokinetics parameters for a drug, which are all significantly associated with the exposure level. Statistical correlations between these pharmacokinetic parameters and treatment outcomes and adverse events were observed. Thus, inter-individual variations in erlotinib pharmacokinetics may present important clinical consequences.

Increasing body of evidences recognized that genetic polymorphisms of drug-metabolizing enzymes and transporters may significantly influence the inter-individual variations in drug reaction and disposition (Evans and McLeod, 2003). As an orally administrated small molecule TKI, erlotinib is mainly metabolized in the liver by cytochrome P450 CYP1A1, 1A2, 3A4, 3A5, and 2D6. Activities of these drug-metabolizing enzymes determine pharmacokinetics of the agents and therefore influence adverse events and therapeutic response rates. Nie et al. have reported that the polymorphism of CYP1A1 is a predictor for clinical outcome in NSCLC patients treated with EGFR-TKI therapy (Nie et al., 2011b). Guillen et al. have also reported that determination of CYP3A4 and CYP1A2 phenotyping in plasma and dried blood spots could predict the pharmacokinetics and toxicity of erlotinib in NSCLC patients (Parra-Guillen et al., 2017). ATP binding cassette subfamily B member 1 (ABCB1) and ATP binding cassette subfamily G member 2 (ABCG2) are important transporter of several drugs, the expression of which appear to affect the pharmacokinetics of erlotinib (Shi et al., 2007; Marchetti et al., 2008; Li et al., 2014). Hamada et al. have reported that ABCB1 1236TT–2677TT–3435TT genotype is associated with higher plasma concentration and the risk of higher toxicity in patients treated with erlotinib (Hamada et al., 2012). Neul et al. have reported that transporter genetic variants are promising novel factors to explain inter-individual variability in the response to TKI therapy (Neul et al., 2016). Chihiro et al. have also reported that the ABCB1 genotype is a potential predictor for erlotinib adverse events (Endo-Tsukude et al., 2018). Although the relationship between SNPs and treatment outcomes and toxicities were widely investigated, the results were inconsistent and none of those analyses clearly explains the determinants of the large inter-individual variability in the treatment outcomes and toxicity of erlotinib treatment.

Accordingly, genetic polymorphisms of the metabolizing enzymes and transporters could affect the expression of corresponding proteins, thus, may predict differences in erlotinib response and their adverse events. To validate this hypothesis, we determined to measure the steady state trough concentration of erlotinb, and analyzed the SNPs in metabolizing enzymes (P450) and transporters (ABC) in NSCLC patients treated with fixed dose erlotinib. We also investigated the correlations between the polymorphisms of these genes with the treatment outcomes and toxicities, respectively.

## Patients and Methods

### Patients and Study Design

The study was carried out at Hunan Cancer Hospital, Hunan, China, between April 2016 and October 2018. Patients with advanced NSCLC who were treated with oral erlotinib at a standard dose of 150 mg were enrolled in our study. Inclusion criteria were as follows: 1) histologically confirmed NSCLC with EGFR sensitive mutation; 2) unmodified dosage of erlotinib during the treatment period. This study was designed and implemented by following the guidelines dictated in the Declaration of Helsinki. Ethics approval was obtained from the Independent Ethics Committee of Hunan Cancer Hospital and all patients were provided written informed consent.

### Erlotinib Trough Plasma Concentration Determination

Blood samples were collected 24 ± 3 h after the last drug administration but before the next administration at day 26-30 after the first dose of erlotinib, which aimed to ensure that the blood samples were steady state trough concentration samples. Blood samples (3 ml each) were collected into EDTA polypropylene tubes, centrifuged at 3000 r/min for 5 min. The remaining samples were prepared for germline mutation detection, while the supernatant in the centrifuged tubes were collected and stored at -80 ℃ until to be analyzed. The plasma concentration of erlotinib was determined by an on-line column extraction HPLC (Shimadzu Corporation, Kyoto, Japan) coupled with binary peak focusing system that we developed. Chromatography was performed as previously described (Liao et al., 2018). The lower limit of quantification was 50 ng/ml for erlotinib.

### Genetic Polymorphism Analysis

The DNA of the patients for genotyping was extracted from peripheral blood according to the method described previously. The genetic polymorphisms of CYP3A4 rs2242480, CYP3A5 rs 776746, CYP2D6 rs 1065852, CYP1A1 rs1048943, CYP1A2 rs762551, rs2470890, ABCB1 rs1128503, rs1045642, ABCG2 rs2231142, rs2622604, rs7699188 were determined by multiple SNP typing techniques. All samples were analyzed in duplicates and negative controls were included to ensure authenticity of the results.

### Data Collection

As typical adverse events, the development of skin rash and diarrhea in all patients were monitored in our study, which were rated according to the National Cancer Institute Common Terminology Criteria for Adverse Events version 4.0 (NCI CTCAE v4.0). Since the median onset of erlotinib induced skin rash and diarrhea are typical within 1–2 weeks of therapy initiation, and toxicities might be confounded by the number of treatment periods, the grade of erlotinib-induced toxicity was determined during the first 30 days. According to NCI CTCAE v4.0, the severity of skin rash was graded as follows: Grade 1: Papules and/or pustules covering <10% body surface area (BSA), which may or may not be associated with symptoms of pruritus or tenderness. Grade 2: Papules and/or pustules covering 10%–30% BSA, which may or may not be associated with symptoms of pruritus or tenderness; associated with psychosocial impact; limiting instrumental activities of daily living (ADL). Grade 3: Papules and/or pustules covering >30% BSA, which may or may not be associated with symptoms of pruritus or tenderness; limiting self care ADL; associated with local superinfection with oral antibiotics indicated. Grade 4: Papules and/or pustules covering any % BSA, which may or may not be associated with symptoms of pruritus or tenderness and are associated with extensive superinfection with IV antibiotics indicated; life threatening consequences. Grade 5: Death. The severity of diarrhea was graded as follows: Grade 1: Increase of <4 stools per day over baseline; mild increase in ostomy output compared to baseline. Grade 2: Increase of 4–6 stools per day over baseline; moderate increase in ostomy output compared to baseline. Grade 3: Increase of ≥7 stools per day over baseline; incontinence; hospitalization indicated; severe increase in ostomy output compared to baseline; limiting self care ADL. Grade 4: Life-threatening consequences; urgent intervention indicated. Grade 5: Death.

Patient characteristic information, such as sex, height, weight, performance status (PS), disease stage (based on the TNM classification), smoking status, mutational status were obtained from their medical records, and progression free survival (PFS) and AEs were strictly assessed and collected by the members in our study after each follow-up visit. Chest computed tomography (CT) was performed just before treatment, besides, chest CT, a complete blood count, and blood chemistry studies were repeated at least once a month until disease progression. Treatment response was assessed using the Response Evaluation Criteria in Solid Tumors version 1.1, and response of treatment was defined as complete response (CR: Disappearance of all target lesions. Any pathological lymph nodes must have reduction in short axis to <10 mm), Partial Response (PR: At least a 30% decrease in the sum of the diameters of target lesions, taking as reference the baseline sum diameters), Progressive Disease (PD: At least a 20% increase in the sum of the diameters of target lesions, taking as reference the smallest sum on study). Stable Disease (SD: Neither sufficient shrinkage to qualify for PR nor sufficient increase to qualify for PD, taking as reference the smallest sum diameters while on study). In addition to the relative increase of 20%, the sum must also demonstrate an absolute increase of at least 5 mm. Follow-up was considered as the duration of treatment under erlotinib. PFS was defined as from the date of erlotinib treatment initiation to the date of first documented progression or death. PFS and adverse event were included in the present analysis to correlate with the mean trough level concentration at steady state and pharmacogenetic factors.

## Statistical Analysis

Data analysis were carried by SPSS version 13.0 software (SPSS Inc., Chicago, IL, USA) in our study and all values were presented as means ± SD. The influence of different genotypes on erlotinib trough levels was analyzed statistically by one-way analysis of variance (ANOVA) with dunnet t test. Fisher's exact tests were used to analyze the association between genetic polymorphisms and toxicity. The patient characteristic factors were tested univariately with Cox regression or Kaplane-Meier analysis. The association between SNPs and PFS was univariately tested with Kaplan-Meier analysis. The SNPs were added to the combined clinical characteristic factors to determine the impact of SNPs on PFS. Statistical significance was assumed for p values < 0.05.

## Results

### Patient Demographics

Patient characteristics at the analysis of the erlotinib plasma concentration were summarized in **Table 1**. A total of 87 advanced NSCLC patients were enrolled in our study, 52 (59.8%) male and 35 (40.2%) female, with a median age of 57 years (29-77). The median body weight was 57 kg (37.0–82.5) and the median body surface area was 1.59 m^2^ (1.24–1.92). All the patients in our study were given a fixed dose (150 mg/d) of erlotinib and had good PS, without dose adjustment. The cohort was consisted of 57 non-smokers, 16 patients with smoking history, and 14 currently-smoking patients. All of the patients had major EGFR mutations, with exon 19 deletions (66.7%) and exon 21 L858R (33.3%) respectively.

**Table 1:** Baseline clinical and pathological characteristics of the 87 patients enrolled in the study.

Patient characteristics	Category/Class	Count (n=24)	Rate (%)
Age (y), median (range)		57(29–77)	
Gender	Female	52	59.8
	Male	35	40.2
Body weight		57(37–82.5)	
BSA (m^2^)		1.59(1.24–1.92)	
Smoking status	Never	57	65.5
	Former	16	18.4
	Present	14	16.1
Clinical stage (UICC)	III	24	27.6
	IV	63	72.4
ECOG PS	0	34	39.1
	1	48	55.2
	2	5	5.7
EGFR mutation status	Exon 19 deletion	58	66.7
	Exon 21 L858R	29	33.3

BSA, body surface area; ECOG PS, eastern cooperative oncology group performance status; EGFR, epidermal growth factor receptor.

### Genotype Frequencies

The genotype status of the drug-metabolizing enzymes and transporters were determined for all 87 patients. The genotype and allele frequency in the study population were presented in **Table 2**.

**Table 2:** Observed genotype and allele frequency of SNPs in the present study (n = 87 patients).

SNP-ID	Gene	Genotype	n	Identified Frequency %	Allele	Allele frequency %
rs2242480	CYP3A4	C/C	40	45.98	C	71.26
		C/T	44	50.57	T	28.74
		T/T	3	3.45		
rs776746	CYP3A5	C/C	30	34.48	C	60.92
		C/T	46	52.87	T	39.08
		T/T	11	12.65		
rs1065852	CYP2D6	A/A	36	41.38	A	60.92
		G/A	34	39.08	G	39.08
		G/G	17	19.54		
rs1048943	CYP1A1	A/A	11	12.64	A	27.01
		A/G	25	28.74	G	72.99
		G/G	51	58.62		
rs762551	CYP1A2	A/A	38	43.68	A	65.52
		C/A	38	43.68	C	34.48
		C/C	11	12.64		
rs2470890	CYP1A2	C/C	74	85.06	C	91.95
		C/T	12	13.79	T	8.05
		T/T	1	1.15		
rs1128503	ABCB1	A/A	40	45.98	A	69.54
		G/A	41	47.12	G	30.46
		G/G	6	6.90		
rs1045642	ABCB1	G/G	34	39.08	G	61.49
		G/A	39	44.83	A	38.51
		A/A	14	16.09		
rs2231142	ABCG2	G/G	48	55.17	G	73.56
		G/T	32	36.78	T	26.44
		T/T	7	8.05		
rs2622604	ABCG2	C/C	52	59.77	C	78.16
		C/T	32	36.78	T	21.84
		T/T	3	3.45		
rs7699188	ABCG2	G/G	77	88.51	G	94.25
		G/A	10	11.49	A	5.75

### Erlotinib Trough Plasma Concentration

Erlotinib trough concentration at steady state was analyzed, and the results were available for 87 patients. The studied NSCLC patients had a wide inter-individual variation in erlotinb trough plasma concentrations, and the mean erlotinib trough plasma concentration was 1354.5 ± 700.3 ng/ml, ranging from 315.6 ng/ml to 4479.83 ng/ml. Trough plasma erlotinib concentration of 8.05% patients were under the target value (500 ng/ml) (Hidalgo et al., 2001), which indicates a high potential risk of disease progression and treatment failure.

### Influence of Smoking Status on Erlotinib Trough Levels

**Figure 1:**
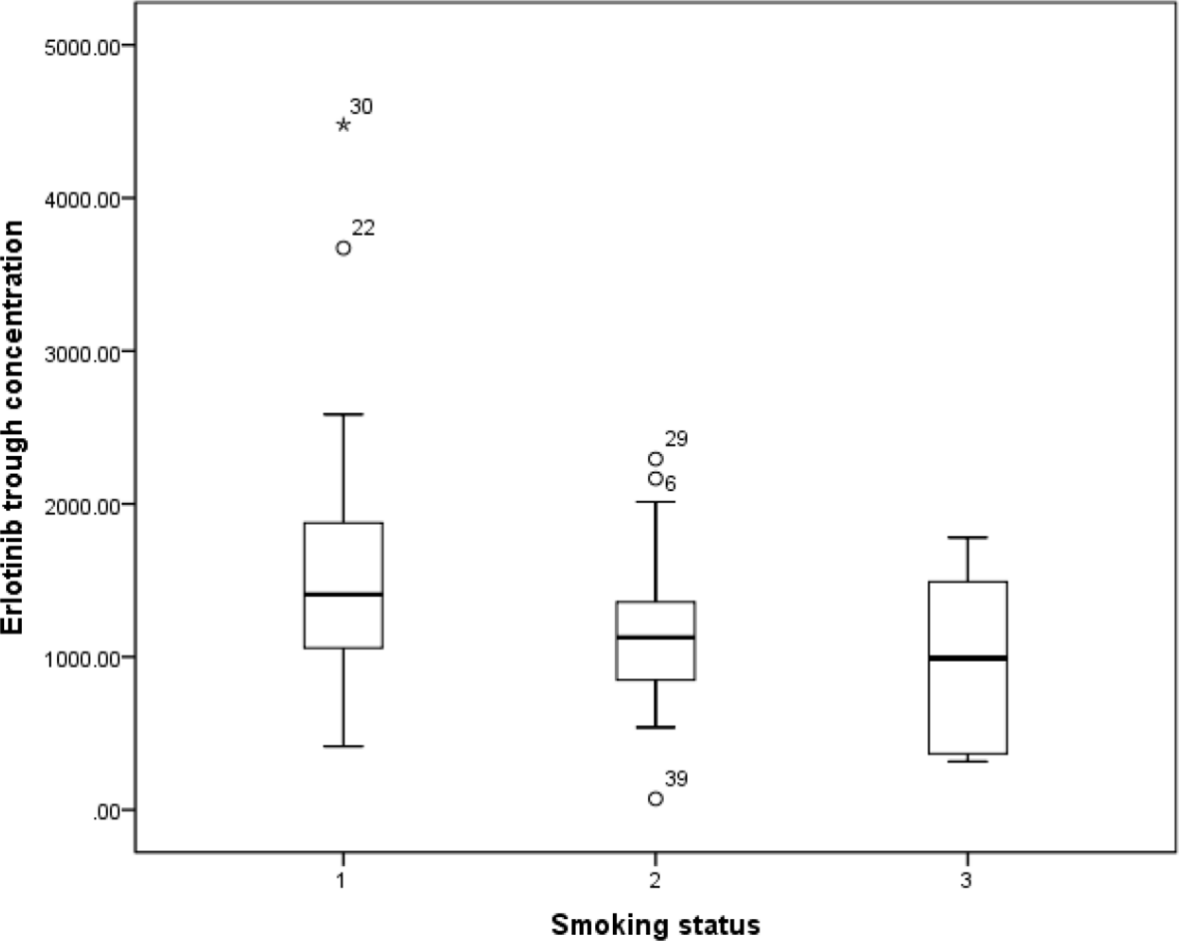
The relationship between erlotinib steady state trough plasma concentrations and smoking status.

Previous study has reported that erlotinib pharmacokinetic was related to smoking status (Hamilton et al., 2006). In our present study, the smoking status for 14 patients was concurrent smoking. The relationship between smoking status and erlotinib steady state trough concentration was investigated, and the results showed that the mean erlotinib trough plasma concentration for concurrent smokers was significantly lower than those without smoking history (p=0.021). The mean erlotinib trough plasma concentration in concurrent smokers was also lower than former smokers, but without statistical difference (**Figure 1**).

### Influence of Different Genotypes on Erlotinib Trough Levels

**Figure 2:**
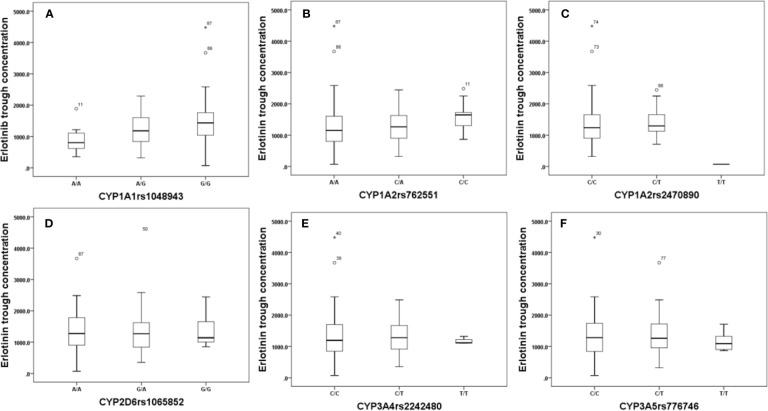
The relationship between erlotinib steady state trough plasma concentrations and CYP1A1rs1048943 **(A)**, CYP1A2rs762551**(B)**, rs2470890 **(C)**, CYP2D6rs1065852 **(D)**, CYP3A4rs2242480 **(E)**, CYP3A5rs776746 **(F)**.

**Figure 3:**
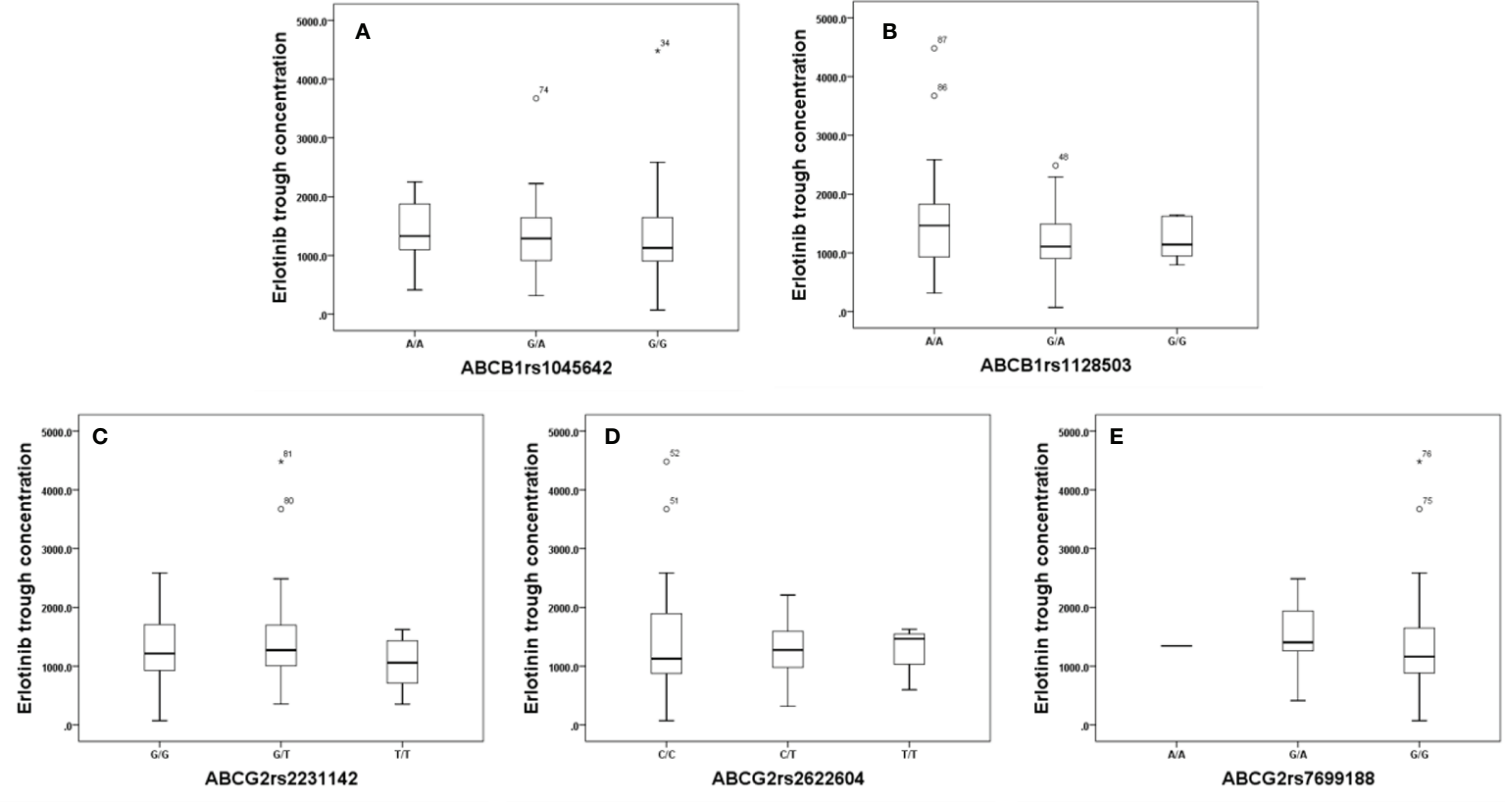
The relationship between erlotinib steady state trough plasma concentrations and ABCB1rs1045642 **(A)**, rs1128503 **(B)**, ABCG2rs2231142 **(C)**, rs2622604 **(D)**, rs7699188 **(E)**.

Effects of genetic polymorphisms of drug-metabolizing enzymes and transporters on erlotinib trough level were investigated in our study. For CYP1A1rs1048943 (**Figure 2A**), the mean steady state erlotinib trough plasma concentration in wild-types (AA) was significantly lower than that of mutant-types (GG) (p=0.007). For CYP1A2rs762551 (**Figure 2B**), rs2470890 (**Figure 2C**), CYP2D6rs1065852 (**Figure 2D**), CYP3A4rs2242480 (**Figure 2E**), CYP3A5rs776746 (**Figure 2F**), no significant difference in the mean steady state erlotinib trough plasma concentration was found in observed genotypes (p > 0.05). In addition, there was no significant difference among the mean trough concentration of patients stratified based on genotypes of all the drug transporters (ABCB1rs1045642, rs1128503, ABCG2rs2231142, rs2622604, rs7699188) (**Figure 3**).

### Effect of Genetic Polymorphisms on Adverse Reactions

As the most frequently observed adverse reactions, the development and severity of skin rash and diarrhea were recorded in our study. As shown in **Table 3**, the severity of skin rash was only associated with the polymorphism of rs762551 in CYP1A2 (p=0.029). For diarrhea, the polymorphisms of rs1045642 (p=0.005) and rs1128503 (p=0.007) in ABCB1 and rs776746 (p=0.048) in CYP3A5 was significantly associated with its development, but not the severity grade. For other metabolizing enzymes and transporters, no significant association was observed between polymorphisms and the development and severity of adverse reactions (p > 0.05).

**Table 3:** Association between genetic polymorphisms and toxicity.

Gene	Polymorphism	Genotype/diplotype	Skin rash (grade)			P	Diarrhea (grade)			P
			0	1	2+		0	1	2+	
ABCB1	rs1045642	G/G	7	20	7	0.614	27	6	1	0.005
		G/A	8	21	10	0.280	25	14	0	0.154
		A/A	1	7	6		4	9	1	
ABCB1	rs1128503	G/G	2	3	1	0.471	4	2	0	0.007
		G/A	8	23	10	0.747	33	7	1	1.000
		A/A	6	22	12		19	20	1	
ABCG2	rs2231142	G/G	9	26	13	1.0	33	14	1	0.554
		G/T	6	18	8	1.0	18	13	1	1.000
		T/T	1	4	2		5	2	0	
ABCG2	rs2622604	C/C	10	26	16	1.0	33	18	1	1.000
		C/T	6	20	6	0.434	21	10	1	1.000
		T/T	0	2	1		2	1	0	
ABCG2	rs7699188	G/G	15	43	19	0.681	49	26	2	1.0
		G/A	1	5	4	0.445	7	3	0	1.0
CYP1A1	rs1048943	C/C	2	5	4	0.680	7	4	0	0.687
		C/T	6	15	4	0.326	18	6	1	1.000
		T/T	8	28	15		31	19	1	
CYP2D6	rs1065852	G/G	2	9	6	0.822	11	5	1	0.958
		G/A	7	16	11	0.200	21	13	0	0.501
		A/A	7	23	6		24	11	1	
CYP3A4	rs2242480	C/C	5	27	8	0.277	21	17	2	0.088
		C/T	11	20	13	0.139	32	12	0	0.275
		T/T	0	1	2		3	0	0	
CYP3A5	rs776746	C/C	3	19	8	0.337	14	14	2	0.048
		C/T	11	25	10	0.264	34	12	0	0.217
		T/T	2	4	5		8	3	0	
CYP1A2	rs762551	C/C	0	7	4	0.300	5	5	1	0.295
		C/A	8	10	20	0.029	24	13	1	0.241
		A/A	8	21	9		27	11	0	
CYP1A2	rs2470890	C/C	13	41	20	0.210	45	17	2	0.634
		C/T	2	7	3	1.000	10	2	0	1.000
		T/T	1	0	0		1	0	0	

First row, grade 0 versus 1+; second row, grade 0–1 versus 2+; p ≤ 0.05 was considered statistically significant.

### Effect of Genetic Polymorphisms on PFS

The relationship between pharmacogenetic factors and PFS was analyzed in our study. In the univariate analysis of PFS, the SNP of rs1048943 in CYP1A1 (AA vs. AG vs. GG, p=0.030) relating to the pharmacodynamics of erlotinib showed an association with survival (**Table 4**). As to the multivariate analysis, the characteristic factors of patient body surface area and smoking status (HR 1.042, P=0.008, HR 5.317, P=0.029, respectively) were associated with shorter PFS. When the SNPs of metabolizing enzymes and transporters were added to this model, it can be observed that the CT allele in rs1048943 was associated with longer PFS (HR 0.226, p=0.038).

**Table 4:** Univariate and multivariate analyses of progression-free survival of EGFR mutated NSCLC with 150 mg erlotinib.

		N patients	Univariate KaplaneMeier analyses			Multivariate Cox regression analyses		
			Median PFS	95% CI	p value	HR	95% CI	p value
Clinical factors								
Age		HR per year increase= 0.979		0.954–1.006	0.128	0.974	0.936–1.012	0.179
Body surface area		HR per year increase= 1.020		1.004–1.036	0.013	1.042	1.011–1.074	0.008
Sex	Male	52	18	13.9–22.1	0.231	1		
	Female	35	15	13.6–16.4		0.375	0.113–1.249	0.110
Smoking status	Never	57	19	15.7–22.3	0.027	1		
	Former	16	16	10.9–22.1		2.529	0.695–9.205	0.159
	Present	14	15	14.1–15.9		5.317	1.184–22.382	0.029
Clinical stage	III	24	24	7.8–40.2	0.204	1		
	IV	63	16	13.4–18.6		2.004	0.769–5.525	0.155
ECOG PS	0	34	15	11.3–18.7	0.203	1		
	1	48	18	15.6–20.4		1.257	0.507–3.115	0.621
	2	5	12	6.8–17.2		3.747	0.579–24.244	0.166
EGFR mutation status	Exon 19 deletion	58	18	15.1–20.9	0.197	1		
	Exon 21 L858R	29	15	11.0–19.0		1.318	0.901–1.927	0.155
Genetic factors								
rs1048943(CYP1A1)	A/A	11	15	8.8–21.2	0.030	1		
	A/G	25	18	16.1–20.0		0.226	0.055–0.923	0.038
	G/G	51	18	13.9–22.1		0.528	0.149–1.869	0.322
rs1065852(CYP2D6)	G/G	36	17	13.2–20.8	0.982	1		
	G/A	34	18	13.4–22.6		0.804	0.358–1.805	0.596
	A/A	17	15	9.5–20.5		1.891	0.756–4.732	0.174
rs2242480(CYP3A4)	C/C	40	17	14.6–19.4	0.802	1		
	C/T	44	15	6.9–23.1		1.532	0.541–4.377	0.422
	T/T	3	15	11.8–18.2		0.742	0.075–7.348	0.799
rs776746(CYP3A5)	C/C	30	18	15.6–20.4	0.831	1		
	C/T	46	15	13.5–16.5		0.649	0.217–1.934	0.437
	T/T	11	23	4.1–41.9		0.313	0.065–1.505	0.147
rs762551(CYP1A2)	C/C	11	15	13.3–16.7	0.097	1		
	C/A	38	18	14.8–21.2		0.784	0.317–1.937	0.597
	A/A	38	31			0.408	0.131–1.266	1.121
rs2470890(CYP1A2)	C/C	74	17	14.4–19.6	0.783	1		
	C/T	12	17	12.9–21.1		1.196	0.435–3.287	0.728
	T/T	1	16			0.092	0.004–2.229	0.142
rs1045642(ABCB1)	G/G	34	16	13.6–18.4	0.857	1		
	G/A	39	19	13.9–24.1		0.935	0.361–2.514	0.923
	A/A	14	17	12.3–21.7		0.659	0.205–2.123	0.485
rs1128503(ABCB1)	G/G	6	18	14.8–21.2	0.158	1		
	G/A	41	15	12.4–17.6		0.764	0.346–1.685	0.504
	A/A	40	13	10.6–15.4		0.920	0.243–3.479	0.903
rs2231142(ABCG2)	G/G	48	16	13.2–18.8	0.598	1		
	G/T	32	20	17.0–23.0		1.297	0.602–2.794	0.506
	T/T	7	15			0.754	0.131–4.352	0.752
rs2622604(ABCG2)	C/C	52	16	13.5–18.5	0.850	1		
	C/T	32	19	14.5–23.5		0.908	0.433–1.905	0.799
	T/T	3	23			2.030	0.426–9.662	0.374
rs7699188(ABCG2)	G/G	77	17	14.8–19.2	0.698	1		
	G/A	10	16	9.3–22.7		0.730	0.226–2.362	0.559

## Discussion

Pharmacokinetics is a potential source of biomarkers and could partially explain the inter-individual variability in erlotinib response in previous studies. In our present study, significant variation of erlotinib steady state trough plasma concentration was observed. The influence of polymorphism of drug metabolizing enzymes and transporters on trough plasma concentration, PFS and adverse events was also observed.

The average erlotinib trough plasma concentration was 1354.5 ± 700.3 ng/ml, ranging from 315.6 ng/ml to 4479.8 ng/ml, with a nearly 14.2 fold variance. Results from our research were consistent with previous studies. Hamada et al. has reported that the mean trough plasma concentration of erlotinib was 1530 ± 780 ng/ml in Japanese NSCLC patient, also with a wide variation (range: 320–3390 ng/ml) (Hamada et al., 2012). Similarly, trough concentration of erlotinib has also been monitored in the study by Nienke and co-workers, in which the mean trough plasma concentrations for erlotinib was 1010 ng/ml, with a nearly 74.0 fold variance, which was larger than our study (Lankheet et al., 2014). 500 ng/ml was recognized as the targeting trough concentration of erlotinib in the treatment of NSCLC (Hidalgo et al., 2001). However, 8.05% of patients have not reached this threshold in our study population, which may lead to a higher risk of disease progression and treatment failure.

The relationship between genetic polymorphisms and erlotinib trough level was also investigated in our study, and the results showed that the mean steady state erlotinib trough plasma concentration in wild-types (AA) of CYP1A1rs1048943 was significantly lower than that of mutant-types (GG) (p=0.007). CYP1A1 is a polymorphic gene which codes for the important phase-I XME aryl hydrocarbon hydroxylase and involves in drug metabolism (Indulski and Lutz, 2000). In addition, rs1048943 is one of the common genetic mutant sites in CYP1A1, which is strongly associated with susceptibility to various cancers (Abd El et al., 2017; Jain et al., 2017). Previous study showed that the polymorphism of CYP1A1 was associated with the treatment outcome of EGFR-TKI in advanced lung cancer patients (Nie et al., 2011b). Samyuktha et al. have also reported that a longer survival rate was observed in those patients who carry the mutant-type gene of CYP1A1 in imatinib treated chronic myeloid leukemia patients (Lakkireddy et al., 2015). The response of imatinib was positively associated with its exposure, which may accounts for the fact that a higher steady state concentration of imatinib was achieved in rs1048943 mutant-type gene patients. Mutant allele GG in rs1048943 is thought to be a risk factor for the development of adverse event, while allele AA in rs1048943 is considered as a risk factor for predicting inadequate clinical efficacy of erlotinib treatment. Therefore, for erlotinib treatment, dose adjustment and drug monitoring are highly suggested in patients who carry allele GG in rs1048943.

The effect of smoking on oral erlotinib pharmacokinetics has been widely studied. In our present study, the steady state trough concentration of erlotinb in concurrent smoking patients was significantly lower than that of those without smoking history, which was consistent with previous studies. Hamilton et al. (2006) have reported that smokers required 300mg of erlotinib to achieve similar C_max_ and area under the curve to those of non-smokers only given a 150 mg dose. Clark et al. (2006) have also reported that steady-state C_trough_ values were about 2-fold lower and the CL/F of erlotinib was about 24% increased in current smokers compared with the values in non-smokers. Investigations into the contribution of CYP1A2 to the metabolism of erlotinib and their induction by cigarette smoking have been widely reported (Hamilton et al., 2006; Anderson and Chan, 2016). Parra-Guillen et al. (2017) have reported that higher CYP1A2 activity was observed in current smoking NSCLC patients. Efficacy of erlotinib in smoking patients following administration of standard doses diminished possibly due to CYP1A2 mediated increased metabolism of the drug in the liver and tumor tissue (Li et al., 2007). However, the effect of smoking on the activity of CYP1A2 was not investigated in our present study, which could be a limitation. In addition, considering that the exact recommendations for the erlotinib dose modification have not been established yet (Hughes et al., 2009), traditional fixed dose (150 mg/d) was chosen as the dosage of erlotinib for concurrent smoking patients, not the increased 300 mg/d in our present study.

It is well established that *in vivo* drug-related therapeutic efficacy and toxicity are strongly associated with genetic factors (Scott, 2011; Patel, 2016). However, few pharmacogenomic biomarkers were used as predictive factor of toxicity for target therapy. To our knowledge, the incidence rates of skin rash and diarrhea were 81.6% and 35.5%, respectively. Findings from our present study showed that the polymorphism of CYP1A2 was the only statically significant covariate responsible for erlotinib induced skin rash (p = 0.029). As previous study showed, CYP1A2 was considerably involved in erlotinib metabolism (Li et al., 2007) and may be of value in predicting the individual metabolizer status of erlotinib (Parra-Guillen et al., 2017). The polymorphism of CYP1A2 may have a significant influence on the pharmacokinetic of erlotinib, which resulted in the variation of the severity of skin rash in our study. However, Chihiro et al. has pointed out that the SNPs in CYP1A2 have no effect on skin rash of erlotinib in Japanese patients, which was contrary to our study (Endo-Tsukude et al., 2018). Other factors additional to CYP1A2 activity may also influence erlotinib induced skin rash. Our present study showed that the occurrence of diarrhea was significantly associated with the tested polymorphisms of the selected genes (ABCB1 and CYP3A5). As a common transporter of erlotinib, ABCB1 was expressed in various organs, especially highly in the entire intestine. Polymorphism of ABCB1 can induce the change in transporter activity, and lower activity leads to higher absorption. Hamada et al. has previously reported that polymorphisms of ABCB1 was prominently related with erlotinib pharmacokinetics and toxicity in NSCLC patients (Hamada et al., 2012). Ma et al. has also reported that several SNPs in ABCB1 were associated with diarrhea in NSCLC patients treated with gefitinib (Ma et al., 2017). Consistent with the studies mentioned above, our present study also observed that SNPs in CYP3A5 were statistically associated with the development of diarrhea. Erlotinib is a substrate of CYP3A5 which is highly and polymorphically expressed (Wojnowski, 2004; Thorn et al., 2005). Charles et al. has also reported that polymorphism of CYP3A5 were accompanied with a higher risk of diarrhea in erlotinib treated NSCLC patients (Rudin et al., 2008). However, the relationship between SNPs in CYP3A5 and diarrhea after administration of erlotinib was rarely reported in other study.

The polymorphisms of CYP1A1 were found to be strongly associated with susceptibility to various cancers (Abd El et al., 2017; Jain et al., 2017). However, little information is known available on the polymorphism of CYP1A1 in relation to the clinical outcomes of NSCLC patients undergoing TKI therapy. Our pharmacogenetic study showed that SNPs in the genes encoding for CYP1A1 were associated with PFS in EGFR sensitive mutation patients who were treated with erlotinib 150 mg/d. The rs1048943 in CYP1A1 were significantly associated with a shorter PFS of patients in the present study. We found that the steady state trough level of erlotinib was significantly increased with this SNP mutation, and previous studies have reported that PFS are positively related with trough concentration in NSCLC patients. Hence, the longer PFS may be induced by the decreased metabolic capacity of CYP1A1 in mutant patients. Nie et al. previously reported that the polymorphism of CYP1A1 has a linkage with clinical outcome of NSCLC patients treated with EGFR-TKI (Nie et al., 2011a; Nie et al., 2011b). Previous study also showed that CYP1A1 genetic polymorphism was a promising predictor to improve chemotherapy effects in patients with metastatic breast cancer (Zhou et al., 2018).

In our present study, SNPs in the pharmacokinetic genes encoding for CYP3A4, CYP2D6 and ABCG2 were not significant associated with erlotinib steady state trough concentration. Furthermore, the same results were also observed for toxicity and survival. Our results were consistent with previous studies (Rudin et al., 2008; Parra-Guillen et al., 2017; Endo-Tsukude et al., 2018), however, it should be pointed out that some results in our study were conflicted with other studies (Rudin et al., 2008; Endo-Tsukude et al., 2018). For the relationship between polymorphisms of CYP3A4, CYP2D6, ABCG2, and survival, a hypothetical explanation would be that most patients had an erlotinib steady state serum level higher than the threshold (500ng/ml) needed for clinical activity, negating any effects that these SNPs may have on the actual serum level above this threshold.

Still, several limitations exist in our study. Firstly, we have only investigated the predictive value of polymorphism of common metabolize enzymes and transporters, the other metabolize enzymes and transporters (such as: organic cation transporters) possibly influencing erlotinib treatment outcomes and toxicity to be explored yet. Secondly, the influence of smoking status on the activity of CYP1A2 was not investigated. Thirdly, the concomitant medication factors with potential PK or pharmacodynamic interactions were not taken into account in our study, which is a limitation of the present study. According to the recorded information of patients in our present study, the incidence rate of concomitantly administered drug was low, which could justify our decision that concomitant medication factors were not considered in the study. However, the effect of concomitant medication factors with potential PK or pharmacodynamic interactions will be considered and investigated in our future study. Last but not the least, a larger sample study is needed to confirm our findings.

## Conclusion

In summary, our results demonstrated that a large variability of erlotinib steady state trough concentration exists in NSCLC patients. It is also observed that SNPs in CYP1A1 may influence the steady state trough concentration of erlotinib and PFS. The polymorphisms of CYP1A2 plays an important role in the severity of skin rash, and the development of diarrhea was associated with SNPs in ABCB1 and CYP3A5. On the basis of these findings, with the aim of improving treatment efficacy and reducing toxicity, it is highly recommended that TDM (therapeutic drug monitoring) and genotyping should be taken into account in an attempt to further individualize treatment of erlotinib in NSCLC.

## Data Availability Statement

The data generated for this study is available to download here: https://www.ebi.ac.uk/ena/data/view/PRJEB37674.

## Ethics Statement

The studies involving human participants were reviewed and approved by Ethics approval was obtained from the Independent Ethics Committee of Hunan Cancer Hospital. The patients/participants provided their written informed consent to participate in this study.

## Author Contributions

NY, DX, and DL designed the study and wrote the protocol. DL, ZL, YZ, DY, NL, and LC performed the experiments and analyzed the data. YC, YF contributed to the reagents and materials. DL drafted the manuscript. NY and DL revised the manuscript content. All authors read and approved the ﬁnal manuscript.

## Funding

This study was supported by National Natural Science Foundation of China (NSFC: 81603206), Hunan Provincial Pharmaceutical Association Fund (NO: hn2017005) and Health and Family Planning Commission Foundation of Hunan Province (grant number B20180252).

## Conflict of Interest

The authors declare that the research was conducted in the absence of any commercial or financial relationships that could be construed as a potential conflict of interest.
